# Enhanced siRNA Delivery and Selective Apoptosis Induction in H1299 Cancer Cells by Layer-by-Layer-Assembled Se Nanocomplexes: Toward More Efficient Cancer Therapy

**DOI:** 10.3389/fmolb.2021.639184

**Published:** 2021-04-20

**Authors:** Maryam Sharifiaghdam, Elnaz Shaabani, Zeynab Sharifiaghdam, Herlinde De Keersmaecker, Riet De Rycke, Stefaan De Smedt, Reza Faridi-Majidi, Kevin Braeckmans, Juan C. Fraire

**Affiliations:** ^1^Department of Medical Nanotechnology, School of Advanced Technologies in Medicine, Tehran University of Medical Sciences, Tehran, Iran; ^2^Laboratory of General Biochemistry and Physical Pharmacy, Faculty of Pharmacy, Ghent University, Ghent, Belgium; ^3^Department of Physiology, School of Medicine, Iran University of Medical Sciences, Tehran, Iran; ^4^Department of Biomedical Molecular Biology, Ghent University, Ghent, Belgium; ^5^VIB Center for Inflammation Research, Ghent, Belgium; ^6^Ghent University Expertise Centre for Transmission Electron Microscopy and VIB BioImaging Core, Ghent, Belgium; ^7^Centre for Advanced Light Microscopy, Ghent University, Ghent, Belgium

**Keywords:** selenium nanoparticle, cancer therapy, siRNA delivery, apoptosis, chitosan, nanomedicine, advanced drug delivery

## Abstract

Nanotechnology has made an important contribution to oncology in recent years, especially for drug delivery. While many different nano-delivery systems have been suggested for cancer therapy, selenium nanoparticles (SeNPs) are particularly promising anticancer drug carriers as their core material offers interesting synergistic effects to cancer cells. Se compounds can exert cytotoxic effects by acting as pro-oxidants that alter cellular redox homeostasis, eventually leading to apoptosis induction in many kinds of cancer cells. Herein, we report on the design and synthesis of novel layer-by-layer Se-based nanocomplexes (LBL-Se-NCs) as carriers of small interfering RNA (siRNA) for combined gene silencing and apoptosis induction in cancer cells. The LBL-Se-NCs were prepared using a straightforward electrostatic assembly of siRNA and chitosan (CS) on the solid core of the SeNP. In this study, we started by investigating the colloidal stability and protection of the complexed siRNA. The results show that CS not only functioned as an anchoring layer for siRNA, but also provided colloidal stability for at least 20 days in different media when CS was applied as a third layer. The release study revealed that siRNA remained better associated with LBL-Se-NCs, with only a release of 35% after 7 days, as compared to CS-NCs with a siRNA release of 100% after 48 h, making the LBL nanocarrier an excellent candidate as an off-the-shelf formulation. When applied to H1299 cells, it was found that they can selectively induce around 32% apoptosis, while significantly less apoptosis (5.6%) was induced in NIH/3T3 normal cells. At the same time, they were capable of efficiently inducing siRNA downregulation (35%) without loss of activity 7 days post-synthesis. We conclude that LBL-Se-NCs are promising siRNA carriers with enhanced stability and with a dual mode of action against cancer cells.

## Introduction

Nanotechnology has made important contributions to oncology in recent years, thanks to its uniquely appealing features for drug delivery, diagnosis, and imaging (Pucci et al., 2019). The use of nanoparticles as drug delivery systems has the potential to overcome some severe adverse effects induced by conventional chemotherapeutic drugs on normal cells. Scientists have been able to control the architecture of nanocarriers in such a way that they can promote local delivery (targeting) and even controlled drug release (Fritz and Lenardo, 2019; Martinelli et al., 2019). According to their design, nanocarriers also have the capability of packaging and protecting cargos that are toxic, fragile, insoluble, or unstable for delivery as free drugs (Saw and Song, 2020).

Nanomaterials, including polymers, lipids, inorganic carriers, polymeric hydrogels, and biomacromolecular scaffolds, have led to the development of systems that can deliver chemotherapeutics to tumor sites for achieving the intended therapeutic effect while minimizing unwanted side effects (Shi et al., 2017; van der Meel et al., 2019). Among the many nanomaterials, selenium nanoparticles (SeNPs) are of increasing interest in cancer therapy. Being an important trace element found in amino acids and proteins, Se is necessary for mammalian life (Oldfield, 2002; Zhang et al., 2009). In the 1960s, it entered the cancer field with the development of Se-based radio labels, like sodium selenite and selenomethionine, for cancer diagnosis (Cavalieri and Scott, 1968; Lenardão et al., 2018). More recently, preclinical studies and human clinical trials have provided support for the preventive and therapeutic roles of Se in cancer development (Wadhwani et al., 2017; Yang et al., 2017; Cui et al., 2018; Zhang et al., 2018). Se is toxic to cancer cells due to a correlated action between its pro-oxidant activity and its ability to generate reactive oxygen species (ROS), which leads to apoptosis (Gandin et al., 2018). Its specificity is conferred by its enhanced uptake and accumulation in cancer cells compared to normal somatic cells (Ruberte et al., 2020). While the reason for this is not completely understood, one of the possible mechanisms is the more reductive environment of the cancer cells, which stimulates the formation of Se–S adducts from Se compounds (Olm et al., 2009). As reported before, Se–S adducts could simulate cystine and mixed disulfides, causing enhanced receptor-mediated uptake of Se in cancer cells (Banjac et al., 2008; Olm et al., 2009; Mandal et al., 2010; Ikram et al., 2021).

It has been reported that SeNPs exhibit higher anticancer efficacy and fewer side effects compared to inorganic and organic selenium-containing molecular compounds (Yu et al., 2012). This has led to the evaluation of SeNPs as carriers of chemotherapeutics such as cisplatin (Li et al., 2013), 5-fluorouracil (Liu et al., 2012), doxorubicin (Huang et al., 2013; Zhou et al., 2017), and irinotecan (Gao et al., 2014). These evaluative studies showed the synergistic effect between the anticancer drugs and selenium, which selectively induced apoptosis.

Apart from the delivery of small molecular drugs, recent studies have confirmed the same synergistic effect of SeNPs in combination with siRNA for targeted gene silencing as well (Li et al., 2016; Chen et al., 2017; Xia et al.,2018a,b, 2020c). siRNA is a synthetic double-stranded RNA that is able to interfere with the expression of specific genes by cleaving the messenger RNA (mRNA). This process is intracellularly mediated by the RNA-induced silencing complex (RISC) (Matzke and Birchler, 2005). siRNA-mediated gene silencing with SeNPs has been investigated for the inactivation of MDR-associated genes (Zheng et al., 2016), HSP70 (Li et al., 2016), and VEGF (Yu et al., 2014). However, the Se delivery systems explored so far presented limitations in terms of the formulation stability and the protection of siRNA. Therefore, the need remains for improvements in the design of Se-based nanocarriers in order to have a more stable formulation while at the same time protecting the siRNA until it reaches the cytosol where it can perform its therapeutic function (Yu et al., 2014; Li et al., 2016; Maiyo and Singh, 2020).

Stability and endosomal escape efficiency of drug delivery systems are directly linked to their physicochemical properties (Lück et al., 1998; Gessner et al., 2002; Thorek and Tsourkas, 2008; Wang et al., 2021), which can be modulated by the use of capping agents (Zhang et al., 2004; Song et al., 2020b). The selection of appropriate capping agents to stabilize SeNPs is regarded as a key requirement for their practical application because aggregation results in a decrease in their bioactivity and bioavailability (Zhang et al., 2012; Zheng et al., 2015). Polysaccharides have been widely used as SeNP capping agents because of their biocompatibility, low toxicity, and biodegradability (Zhang et al., 2004; Yu et al., 2012). In that sense, chitosan (CS), which is composed of randomly distributed β-(1→4)-linked D-glucosamine (deacetylated unit) and *N*-acetyl-D-glucosamine (acetylated unit) (Chen and Park, 2003; Kumar et al., 2004), is one of the most widely used naturally biocompatible and biodegradable polysaccharides. Due to its cationic nature, using CS as a capping agent should allow for the electrostatic attachment of negatively charged siRNA molecules to their surfaces (Han et al., 2012; Ahmad et al., 2021; Shaabani et al., 2021).

**SCHEME 1:**
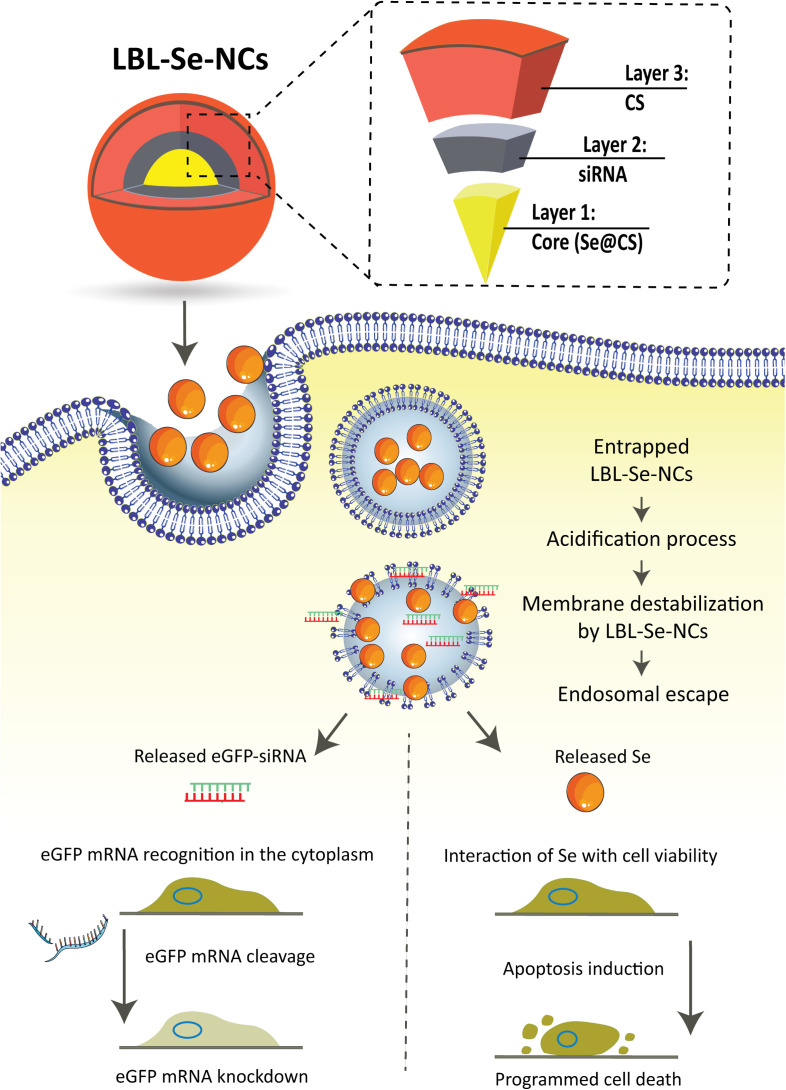
Schematic illustration of layer-by-layer selenium nanocomplexes (LBL-Se-NCs). LBL-Se-NCs are composed of three layers: the core (*Se@CS*), a second layer with the cargo (*siRNA*), and a third protective layer of chitosan (*CS*). The nanocomplexes are taken up by cancer cells *via* endocytosis and trafficked *via* endosomes before finally escaping from endolysosomes. The released eGFP-siRNA and Se will induce a dual effect of apoptosis on the one hand and knockdown of the target protein on the other hand.

In the present study, we used a straightforward layer-by-layer self-assembly approach to synthesize stable Se-based siRNA-loaded nanocomplexes (NCs). SeNPs are first capped with chitosan during their synthesis to obtain Se@CS nanoparticles with excellent colloidal stability. Next, siRNA is electrostatically attached in the second step, forming Se@CS:siRNA nanoparticles. In the final step, the final layer of chitosan is applied (Se@CS:siRNA:CS) to provide protection to the siRNA and to create a net cationic surface charge for better cellular uptake (Scheme 1). These layer-by-layer Se nanocomplexes (LBL Se-NCs) were characterized in terms of their size, surface charge, stability, siRNA-binding capacity, and siRNA release profile. Next, their *in vitro* uptake, cytotoxicity, and apoptotic effect on H1299 lung carcinoma cancer cells were investigated in comparison to siRNA-loaded nanocomplexes prepared from chitosan alone (CS-NCs). The selective anticancer effect of LBL-Se-NCs was investigated by including NIH/3T3 fibroblasts in the study as a normal cell line, similar to previous reports (Ye et al., 2017). Overall, it was found that LBL-Se-NCs have good colloidal stability and provide protection to siRNA while being able to selectively induce apoptosis and siRNA gene silencing in cancer cells. As such, this study is an important step forward to unlocking the potential of Se-based carriers for cancer gene therapy.

## Experimental Section

### Materials

Na_2_SeO_3_ and chitosan (low molecular weight, degree of deacetylation: 80%) were purchased from Sigma-Aldrich (St. Louis, MO, United States). RPMI-1640, L-glutamine, Penicillin/Streptomycin solution (5,000 IU/ml penicillin and 5,000 μg/ml streptomycin) (P/S), Fetal Bovine Serum (FBS), Trypan Blue, 0.25% Trypsin-Ethylenediaminetetraacetic acid (EDTA), and Dulbecco’s phosphate-buffered saline (DPBS) were supplied by Gibco BRL (Merelbeke, Belgium). CellTiter-Glo^®^ Luminescent Cell Viability Assay was purchased from Promega (Leiden, Netherlands). Hoechst 33342 was purchased from Molecular Probes (Erembodegem, Belgium). Lipofectamine RNAiMAX reagent was purchased from Invitrogen (Carlsbad, CA, United States). jetPEI (jetPRIME^®^) was purchased from Polyplus-transfection^®^ company (France).

Small interfering RNA against eGFP (sieGFP, sense strand: *5′-CAAGCUGACCCUGAAGUUCtt-3′*; antisense strand: *GAACU UCAGGGUCAGCUUGtt-3′*) and non-targeted siRNA (siCTRL, sense strand: *5′-CAAGCUGACCCUGAAGUUCtt-3′*; antisense strand: 5′-*GAACUUCAGGGUCAGCUUGtt-3′*) were purchased from Eurogentec (Seraing, Belgium). For the uptake experiments, the siCTRL duplex was labeled with Alexa Fluor 647 dye at the 5′ end of the sense strand (si-AF647) (Eurogentec). For the endosomal escape, red-labeled fluorescent oligonucleotides (AF647 ONs) were used (Eurogentec).

In this study, we used H1299 and NIH/3T3 cells with a passage number below 20. The number of cells was 7,500 cells/ml and 9,000 cells/ml for the 24 and 48 h studies, respectively.

### Preparation of NCs

#### Core Nanoparticle Synthesis: Se@CS

##### Se@CS

Se nanoparticles stabilized with chitosan were prepared as previously reported with minor modifications (Boroumand et al., 2019). Briefly, 4 ml of 200 mM vitamin C (ascorbic acid) solution was added drop-wise into 5.83 mg Na_2_SeO_3_ dissolved in 40 ml of 0.2% (w/v) chitosan solution under magnetic stirring at 900 RPM for half an hour at room temperature. The stabilized Se@CS nanoparticles were centrifuged at 21,000 *g* for 60 min allowing the collection of the Se@CS NPs while removing the remaining excess materials. The collected NPs were dispersed in RNase-free water. UV-Visible, dynamic light scattering (DLS)/Zeta potential, and Fourier transform infrared spectroscopy (FTIR) characterization were performed to confirm the synthesis of the core nanoparticles (Se@CS).

#### Layer-by-Layer Selenium Nanocomplex Synthesis: LBL-Se-NCs

The LBL-Se-NCs are composed of three layers: the core (Se@CS), a second layer with the siRNA (*Se@CS:siRNA*), and a third protective chitosan layer (*Se@CS:siRNA:CS*). These LBL-Se-NCs were synthesized in two steps, given as follows:

##### Se@CS:siRNA

Surface loading of siRNA was carried on different weight ratios of siRNA to Se from 1:2.5 to 1:40 (w/w) under constant stirring at 400 RPM for 60 min.

##### Se@CS:siRNA:CS

Finally, after 60 min, the third layer was applied by adding a 0.5% (w/v) chitosan solution to the reaction under continuous stirring for another 60 min. The resulting NCs were centrifuged again at 21,000 *g*/min for 60 min to remove all unreacted materials, after which the collected precipitate was resuspended in RNase-free water.

#### CS-NCs

In this study, chitosan is the most determining part with regard to siRNA complexation, cellular uptake, and endosomal release; so, we consider CS-NCs as a suitable control group to compare with the Se@CS:siRNA:CS group. In this regard, siRNA-chitosan nanocomplexes were prepared by the ionic gelation method, reported in a prior study, with some modifications (Katas and Alpar, 2006). CS-NCs were prepared by adding siRNA in double-distilled water to the tripolyphosphate (TPP) solution (1.2 ml, 1 mg/ml) before adding this drop-wise to the chitosan solution (3 ml, 2 mg/ml) under constant magnetic stirring at room temperature. The particles were then incubated at room temperature for another 30 min before centrifugation at 21,000 *g* at a temperature of 4°C.

### Characterization of NCs

Hydrodynamic diameter and surface charge of LBL-Se-NCs were determined using a Zetasizer Nano (Malvern, Worcestershire, United Kingdom) in each step of the synthesis.

The molecular structure of the core nanoparticles (Se@CS) was analyzed using FTIR by mixing the core nanoparticles with KBr (1:50, w/w) and pressing them into uniform pellets to perform scanning in the wavenumber range 4,000–400 cm^–1^.

Transmission electron microscopy (TEM) images were obtained at the VIB-UGent Transmission Electron Microscopy-Core facility using a JEM 1400 plus transmission electron microscope (JEOL, Tokyo, Japan) operating at 80 kV. Samples were prepared for the TEM analysis by adding 50 μl of the nanoparticle dispersions on formvar/C-coated hexagonal copper grids (EMS G200H-Cu). After 20 min of waiting at room temperature, they were washed with double-distilled water several times.

The entrapment of siRNA in NCs was assessed by a NanoDrop 2000c spectrophotometer (Thermo Scientific, Rockford, IL, United States). To determine the amount of free siRNA in the supernatant recovered after particle centrifugation (21,000 × *g*, 60 min), the typical absorbance of nucleic acids at 260 nm was monitored. siRNA entrapment in NCs was further confirmed by agarose gel electrophoresis. A 2% agarose gel was prepared by dissolving agarose (UltraPure Agarose, Invitrogen, Erembodegem, Belgium) in a 100 ml TBE buffer (98 mM Tris, 88 mM Boric acid, 2 mM Na_2_EDTA with pH 8). GelRed (Biotium, Hayward, CA, United States) was added to the TBE buffer for siRNA detection and 5 μl of 5 × Gel Loading Buffer (Ambion, Merelbeke, Belgium) was added for every 20 μl of the sample before running for 20 min at 120 V. A total volume of 20 μl was pipetted per lane and a siRNA ladder was added as well as a control. We used a Kodak digital science camera (Kodak EDAS 120, Rochester, NY, United States) to take an image of the gel under UV light (Bio-Rad UV Transilluminator 2000, CA, United States).

### Stability of NCs

The stability of the LBL-Se-NCs was determined after resuspending and incubating NCs in different media (ddi. water pH 7.4, DMEM [Dulbecco’s Modified Eagle’s Medium], HEPES, PBS, acidic water [pH 3], and alkaline water [pH 9]) for 1 h and observing changes in the hydrodynamic diameter. The stability of the NCs was also evaluated by monitoring changes in the hydrodynamic diameter and zeta potential in RNase-free water at room temperature for both LBL-Se-NCs and CS-NCs over 20 days.

### siRNA Release From the NCs

The *in vitro* release study was carried out by determining the released siRNA from LBL-Se-NCs and CS-NCs post-synthesis. In this technique, we separated the release media from the nanoparticles by centrifugation (Cetin et al., 2010; D’Souza, 2014). The released siRNA was quantified in the supernatant using a NanoDrop at a wavelength of 260 nm. Briefly, LBL-Se-NCs and CS-NCs were incubated in microtubes containing 10 mM HEPES buffer with a pH of 7.4 and gently shaken at 37°C. At specific time points, the supernatant of the samples was taken from one of the microtubes after centrifuging at 21,000 *g* for 60 min, and the concentration of siRNA in the supernatant was measured to determine the amount of released siRNA at each time point during 7 days after synthesis.

### Cytotoxicity Studies

H1299 cells (lung epithelial cells derived from metastatic lymph nodes, ATCC-CCL 5803) stably expressing enhanced green fluorescence protein (eGFP) were cultured in Roswell Park Memorial Institute (RPMI) 1640 medium supplemented with 10% FBS, 2 mM L-Glutamine, and 100 μg/ml P/S in a humidified incubator at 37°C with 5% CO_2_ atmosphere. NIH/3T3 cells (embryo-derived fibroblast cells, ATCC-CRL-1658) were cultured in DMEM supplemented with 10% Calf serum, 2 mM L-Glutamine, and 100 μg/ml P/S in a humidified incubator at 37°C with 5% CO_2_ atmosphere. For all *in vitro* studies, NCs, prepared at a 1:40 weight ratio of siRNA to Se, were diluted in a culture medium to reach a final concentration of 2–64 nM siRNA.

The cells were seeded in 96-well plates at 7,500 cells per well (for 48 h study) and were allowed to attach overnight. On the next day, the cells were treated with the prepared NCs (2–64 nM siRNA concentration) for 4 h at 37°C in cell culture medium. Next, the cells were washed once with PBS and incubated in a fresh cell medium for an additional 24 h before they were prepared for measuring their metabolic activity by the CellTiter-Glo^®^ assay. On the next day, after replacing the cell culture medium with 100 μl of fresh medium, 100 μl of CellTiter-Glo^®^ reagent was added in each well and mixed for 20 min on an orbital shaker (Rotamax 120, Heidolph, Germany) to induce cell lysis. The mix was further incubated at room temperature for 10 min to let the luminescent signal stabilize. Next, 100 μl suspension of each well was transferred to white 96-well plates and the luminescence signal was recorded by a GloMax^®^ 96 Luminometer (Promega). Wells containing medium without cells were included to determine the background luminescence.

### DiIC_1_(5)/Propidium Iodide Apoptosis Assay

Induction of apoptosis in NCs-treated H1299 and NIH/3T3 cells was evaluated by flow cytometry using propidium iodide (PI) and dihexaoxacarbocyanine iodide (DiIC_1_). The cells were seeded in a 96 well culture plate and cultured for 24 h, after which they were treated with different concentrations of LBL-Se-NCs and CS-NCs (2–64 nM siRNA concentration) for 4 h at 37°C in a cell culture medium. Next, the cells were washed and incubated for an additional 24 h. On the next day, the culture medium was removed and transferred to another plate because the removed medium could contain dead cells that should also be included in the analysis. The cells were detached from the well plates using trypsin and then neutralized after 5 min with the medium that was kept from the previous step (and that may contain additional dead cells). Following centrifugation (5 min, 500 *g*), the cell pellet was resuspended in staining buffer prepared from flow buffer (DPBS^–^, 0.1% Sodium Azide, 1% Bovine Serum Albumin) to which PI (1 μg/ml) and DiIC_1_ (10 nM) were added. The cells were incubated with the staining buffer for 30 min at 37°C before being analyzed with flow cytometry (CytoFLEX, Beckman Coulter, Krefeld, Germany). The FlowJo software (Tree Star Inc., Ashland, OR, United States) was used to separate the cell population into three parts: Q1: live cells (high intensity of deep red fluorescence, DilC_1_(5)^+^/PI^–^); Q3: apoptotic cells [no fluorescence, DilC_1_(5)^–^/PI^–^]; and Q4: dead cells [red fluorescence, DilC_1_(5)^–^/PI^+^].

### Evaluation of Uptake via Flow Cytometry

Uptake experiments were performed by seeding 9,000 H1299 and NIH/3T3 cells per well in 96-well plates which were allowed to attach overnight. The next day, NCs containing AF647 siRNA were prepared as described above. The cells were incubated with LBL-Se-NCs and CS-NCs (2–64 nM siRNA concentration) for 4 h at 37°C, after which they were washed and detached from the well plates using trypsin, and then neutralized with a complete cell culture medium. The cells were transferred to U-Bottom 96-well (Greiner Bio-One GmbH, Vilvoorde, Belgium) plates and centrifuged (5 min, 500 *g*), after which the cell pellet was resuspended in flow buffer for flow cytometry analysis.

For each sample, the forward and side scatter (FSC and SSC, respectively) were measured for 60 s at a flow rate of 60 μl/min. Red fluorescence (excitation at 638 nm and detection with a 660/20 nm bandpass filter) was measured using a CytoFLEX flow cytometer. Finally, data analysis was performed using the FlowJo software for quantifying the percentage of positive cells (uptake [%]) as well as the relative mean fluorescence intensity per cell (rMFI) according to:

rMFI⁢(relative⁢Mean⁢Fluorescence⁢Intensity)=MFI⁢of⁢cells⁢treated⁢with⁢Alexa⁢Fluor⁢ 647⁢siRNAMFI⁢of⁢cells⁢treated⁢with⁢nonlabeled⁢siRNA

### Evaluation of Transfection Efficiency via Flow Cytometry

For calculating the siRNA gene silencing efficiency on eGFP-H1299 cells, 7,500 cells per well were seeded (for 48 h study) in 96-well plates which were allowed to attach overnight. On the next day, the cells were treated with NCs containing eGFP-siRNA (sieGFP) and negative control-siRNA (siCTRL) (2–64 nM siRNA concentration) for 4 h at 37°C in RPMI. Next, the cells were washed and incubated for an additional 24 h. On the next day, following the same steps previously mentioned for flow cytometry, the cells were washed, detached, centrifuged, and resuspended in flow buffer for flow cytometry analysis. For each sample, the forward and side scatter (FSC and SSC, respectively) were measured for 60 s at a flow rate of 60 μl/min. The samples were excited with a 488 nm laser line and the signal was detected with a 525/40 filter using a CytoFLEX plate reader flow cytometer. The FlowJo software was used to measure eGFP expression according to the following equation:

$$GFPExpression\left(\%\right)=\frac{M\,F\,I\,s\,i\,G\,F\,P}{M\,F\,I\,s\,i\,C\,T\,R\,L}\times 100$$

*MFI siGFP* indicates the mean fluorescence intensity of cells treated with GFP-siRNA *and MFI siCTRL* indicates the mean fluorescence intensity of cells treated with negative control-siRNA.

The eGFP knockdown efficacy was also evaluated by taking microscopy images after treatment with a Nikon A1R HD confocal laser scanning microscope (Nikon Benelux, Brussels, Belgium), equipped with a laser box (LU-N4 LASER UNIT 405/488/561/640, Nikon Benelux, Brussels Belgium), detector box (A1-DUG-2 GaAsP Multi-Detector Unit, GaAsp PMT for 488 and 561 and Multi-Alkali PMT for 647 and 405 nm), and a 20X air objective lens (CFI plan Apo VC 20X, NA 0.75, WD 1,000 μm) (Nikon, Japan). The images were recorded using the NIS Elements software (Nikon, Japan). Briefly, H1299-eGFP cells were seeded in 35 mm CELLview microscopy dishes with glass bottom (Greiner Bio-One, Vilvoorde, Belgium) at a density of 75,000 cells per ml and were allowed to settle overnight. After removal of cell culture medium, the cells were treated for 4 h with LBL-Se-NCs, jetPEI, Lipofectamine, and CS-NCs, all containing 8 nM eGFP-siRNA and 8 nM naked eGFP-siRNA as a carrier-free control. Live-cell imaging was performed for evaluating knockdown efficacy after 24 h of treatment at 37°C. Before confocal imaging, the cells were washed and stained with Hoechst 33342 (1 mg/ml in H2O; 1,000 × diluted). After 20 min of incubation with Hoechst, the cells were washed three times with PBS. The stained cells were provided with a full cell culture medium and put on the microscope with an incubator to enable live-cell imaging at optimal environmental conditions (5% CO_2_, 100% humidity, and 37°C). The 408 and 488 nm laser lines were used to excite the Hoechst-labeled nuclei and the eGFP protein, respectively.

### Visualization and Quantification of Endosomal Escape

Endosomal escape was evaluated by a dequenching assay first published by Rehman et al. (2013). For this, LBL-Se-NCs, jetPEI, Lipofectamine, and CS-NCs containing 8 nM AF647 ONs (red-labeled fluorescent oligonucleotides) were prepared and added to cells (75,000 cells per ml seeded in 35 mm CELLview microscopy dishes [Greiner Bio-One, Vilvoorde, Belgium]) for 4 h at 37°C, after which they were washed and incubated overnight at 37°C and 5% CO_2_. After 24 h, the cell nuclei were stained with Hoechst 33342 (1 mg/ml in H_2_O; 1000 × diluted) and incubated for 20 min 37°C and 5% CO_2_. Immediately after washing, the cells were provided with a full cell culture medium and were placed in the incubator of the microscope (37°C under a humidified 5% CO_2_ atmosphere).

For visualization, the 408, 488, and 633 nm laser lines were applied to excite the Hoechst-labeled nuclei, eGFP protein, and the AF647 ONs, respectively. Upon endosomal escape, free AF647 ONs would be released into the cytosol and accumulate into the nucleus. Data analysis with the ImageJ (FIJI) (Schindelin et al., 2012) software was performed to determine both the total number of cells and the number of cells with AF647 ON-positive nuclei. The ratios of both give the percentage of cells in which at least one endosomal escape event has happened. The nuclei were detected in the blue channel by thresholding (applying the same offset values for every image), and intensity analysis of the nuclear fluorescence signal in the red channel was performed. At least 250 cells in a minimum of 25 images were included for all conditions tested.

### Statistical Analysis

All statistical analyses were performed using the GraphPad software (La Jolla, CA, United States), and the propagation of errors was applied when necessary. One-way ANOVA or the Student’s *t*-test was used for comparing conditions. All results are reported as mean ± SD and differences with a *p*-value < 0.05 considered significant.

## Results

### Core (Se@CS) Nanoparticle Synthesis and Characterization

**FIGURE 1:**
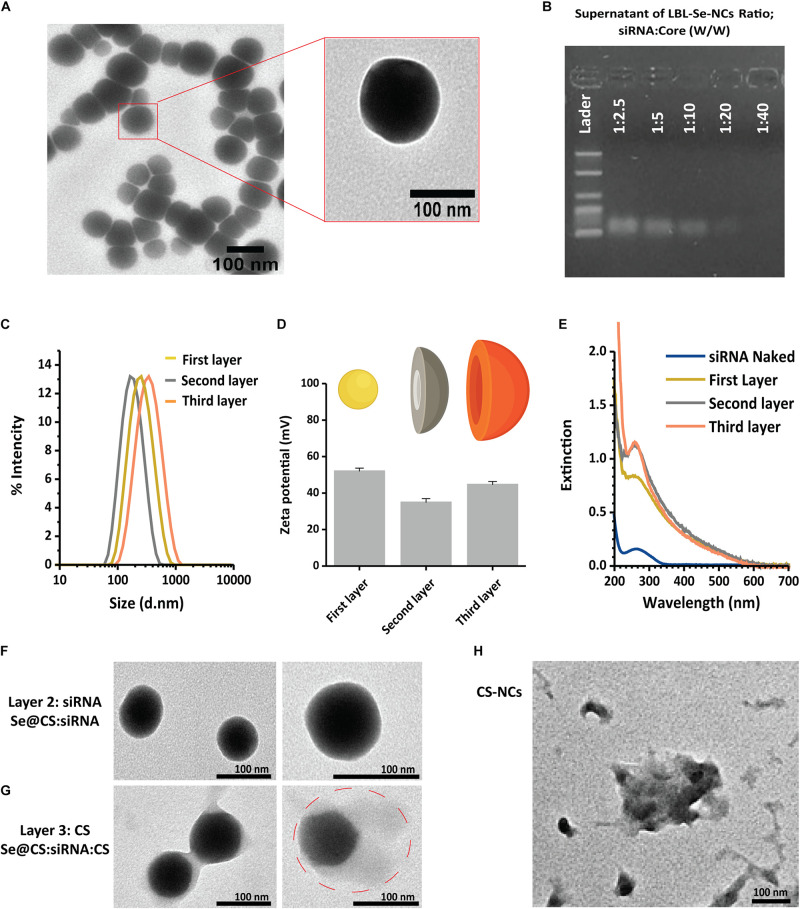
Synthesis of LBL-Se-NCs. **(A)** TEM images of Se@CS nanoparticles. The images confirmed the formation of SeNPs and indicate an average particle size of around 74.3 ± 5 nm. **(B)** The siRNA-loading capacity of Se@CS nanoparticles, from 1:2.5 to 1:40 siRNA:core mass ratios, was evaluated by measuring the remaining siRNA content in the supernatant by agarose gel electrophoresis. **(C,D)** The hydrodynamic diameter **(C)** and the surface charge **(D)** of the nanoparticles were evaluated with dynamic light scattering after each step in the synthesis process. The first layer corresponds to Se@CS, the second layer to Se@CS:siRNA, and the third layer to Se@CS:siRNA:CS. (*n* = 3). **(E)** UV-Visible spectra of the different stages of the LBL synthesis of Se-NCs. **(F)** TEM images of Se@CS:siRNA **(G)** and Se@CS:siRNA:CS. **(H)** TEM images of CS-NCs.

The main goal of this study is the design and synthesis of novel layer-by-layer Se-based nanocomplexes (LBL-Se-NCs) for siRNA delivery in synergy with the native anticancer properties of selenium. For this, we started by synthesizing core SeNPs using chitosan as a coating (Se@CS). TEM imaging confirmed the formation of Se nanoparticles with a size of 74.3 ± 5 nm (Figure 1A). The hydrodynamic diameter of the Se@CS core nanoparticles was measured in ddi. water as well, showing an average diameter of 218 ± 3 nm with PDI = 0.24 and a positive Zeta potential of 52 ± 0.1 mV which suggested that the chitosan was successfully capped on the surface of the nanoparticle (Figures 1C,D, ‘first layer’). The hydrodynamic size as measured by DLS being larger than what is measured by TEM is likely due to the fact that in DLS, the polymer coating of the particle is fully hydrated, while the samples are dehydrated for TEM imaging. To further prove the presence of a chitosan coating, the Se@CS nanoparticles were additionally analyzed by FTIR. The spectrum shown in Supplementary Figure 1A has clear features at 3,300–3,500 cm^–1^ which correspond to the vibrational modes of chitosan, in particular, stretching vibrations of O-H and N-H. Compared to the spectrum of chitosan alone, there is a slight blue shift due to the formation of hydrogen bonds between selenium and chitosan (Song et al.,2020a,b). Other clear characteristic bands of chitosan can be seen at 1,664 (amide I), 1,548 (amide II), and 1,407cm^–1^ (amide III). The peak located at 1,150 cm^–1^ is the result of chitosan deacetylation and is related to asymmetric vibrations of CO in the oxygen bridge. The absorption bands at 1,082 and 1,032 cm^–1^ (skeletal vibrations involving the C-O stretching) are characteristic of its saccharide structure (Silva et al., 2012; Yu et al., 2012; Song et al., 2020b). These vibrational modes present in the FTIR spectra further confirmed the presence of CS on the surface of the core nanoparticles.

### Formation and Characterization of LBL-Se-NCs

The next step was to load siRNA on the Se@CS nanoparticles. siRNA was added at different weight ratios of siRNA to Se from 1:2.5 to 1:40 (w/w). After the addition of siRNA to the Se@CS core particles, the dispersion was centrifuged to remove any unbound siRNA. The presence of unbound siRNA in the supernatant was investigated by UV-Vis spectroscopy and agarose gel electrophoresis in order to determine the siRNA-loading capacity of the Se@CS core nanoparticles. When performing agarose gel electrophoresis on the supernatant, it was found that siRNA was fully complexed to the core nanoparticles at a ratio of 1:40, corresponding to an effective siRNA concentration of 160 nM (Figure 1B). At higher ratios, an increasing amount of unbound siRNA was detected in the supernatant. This was further confirmed by the UV-Vis spectra of the supernatant (Supplementary Figure 1B), which showed a gradual increase in unbound siRNA as the ratio increased.

Based on these results, LBL-Se-NCs at a ratio of 1:40 (w/w) with an effective siRNA concentration of 160 nM were selected for further characterization. Upon coating with anionic siRNA, the positive surface charge of Se@CS decreased as expected, reaching a value of 35.0 ± 1.5 mV. At the same time, a decrease in the hydrodynamic diameter was observed from 218 ± 3 nm (Se@CS) to 174 ± 1.2 nm (Se@Cs:siRNA) (Figures 1C,D). The reason for this decrease can be related to the condensation state of the polymer by the incorporation of small and highly negatively charged molecules in the macromolecular organization of the LBL structure (siRNA). Finally, the last protective chitosan layer was applied, thus obtaining Se@CS:siRNA:CS nanocomplexes, whose zeta potential increased again to 44 ± 3 mV, with a size of 237.0 ± 0.8 nm (Figures 1C,D).

The process of layer coating on the core was also monitored by UV-Vis spectroscopy. As shown in Figure 1E the synthesized Se@CS had a band with a maximum of about 260 nm. However, after the deposition of siRNA on the core surface to form Se@CS:siRNA, the intensity of the band increased, indicating the formation of the siRNA layer. In addition, after the formation of the third layer with chitosan, an increase of around 200 nm at the extinction spectrum was observed, which is related to the attached chitosan on the surface.

We performed additional experiments in which the stability of Se@CS:siRNA nanocomplexes was examined after purification by centrifugation before and after applying the third chitosan layer. Analysis by DLS and simple visual inspection clearly showed that the presence of the third layer has an effect on the resuspension of the NCs in water. For nanocomplexes without the third layer, we could observe flocculation and sedimentation, while DLS also showed that the overall size of the NCs had increased with clear signs of aggregation (Supplementary Figures 1C,D).

The TEM images were also recorded after the second (Se@CS:siRNA) and the third step (Se@CS:siRNA:CS). The addition of the second siRNA (Figure 1F) did not change the morphology or size with sizes of 74 ± 5 and 73 ± 8 nm for the core post-synthesis and for the complexes after attachment of the siRNA (second layer), respectively. Despite observing a reduction in size after the attachment of the second layer in the DLS study, this change in size was not visible in the microscopic images. The reason why this difference is better detectable by DLS could be the expansion of the polymer coating in the aqueous phase during DLS measurements, while TEM imaging is performed on dehydrated samples. Also, similar changes in size are reported in the study by Maiyo and Singh (2020) in which, after the addition of siRNA on CS-covered Se nanoparticles, the nanocomplexes had slightly reduced diameters compared to uncomplexed nanoparticles.

On the other hand, the final chitosan layer was visible in the TEM images as a faint ‘halo,’ which resulted in an effective increase of the overall size of the nanoparticle to 95 ± 11 nm (Figure 1G). Figure 1H is TEM image of CS-NCs, and as can be seen, their solidarity is less than LBL-Se-NCs.

### Evaluation of Stability of LBL-Se-NCs and siRNA Release Profile

**FIGURE 2:**
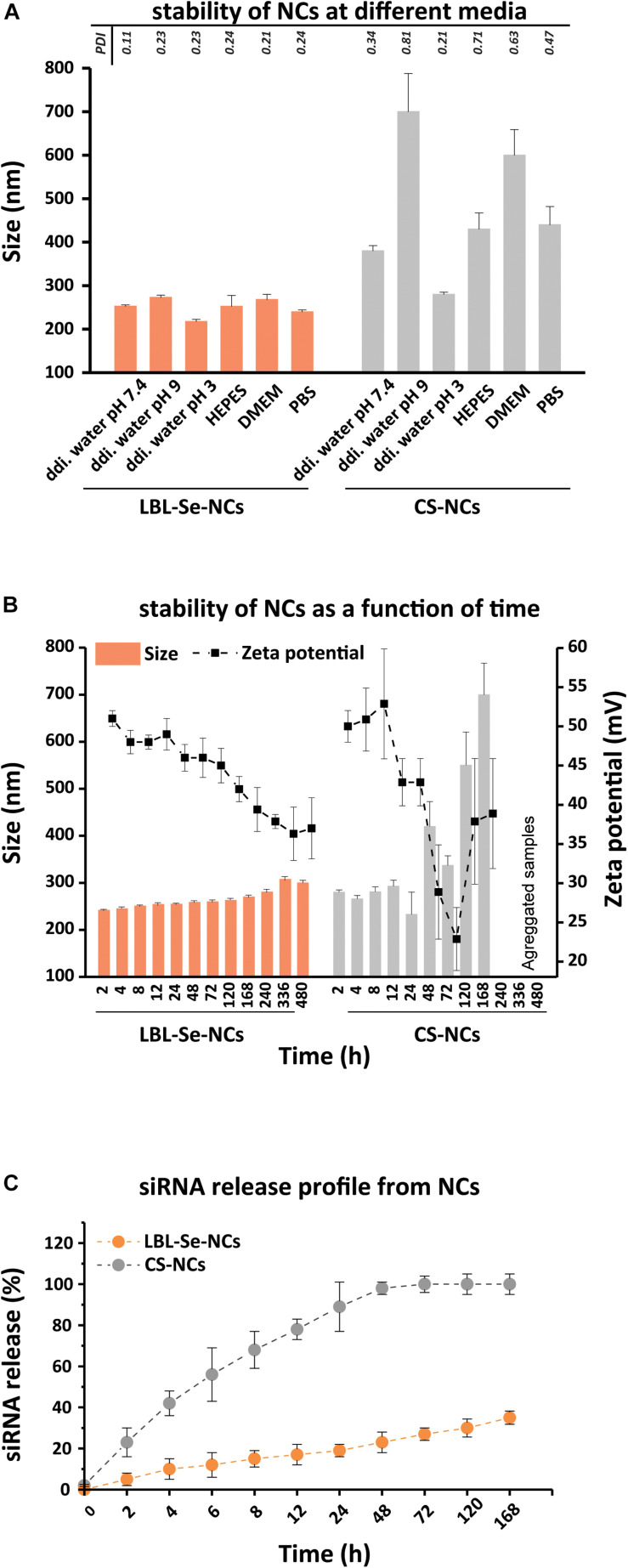
Evaluation of LBL-Se-NC colloidal stability and siRNA release. Characterization of LBL-Se-NCs and CS-NC **(A)** colloidal stability in different media: ddi. water (pH 7.4, 3, 9), DMEM, HEPES, PBS. **(B)** Colloidal stability as a function of time in RNase-free water as measured by DLS and zeta potential. **(C)** siRNA release profile from LBL-Se-NCs and CS-NCs as a function of time. Bars represent mean ± SEM, for a minimum of three independent experiments.

One of the first challenges for a drug delivery system is achieving good colloidal stability and not preventing aggregation (Buyens et al., 2008, 2012; Raemdonck et al., 2013). Therefore, we first evaluated the stability of LBL-Se-NCs in different media, including ddi. water (pH 7.4, 3, 9), HEPES buffer, DMEM, and PBS. To judge the role of the Se core, a comparison was performed with nanocomplexes prepared from CS-NCs. Figure 2A shows the hydrodynamic size of both LBL-Se-NCs and CS-NCs after 1 h of incubation in the different media. It is immediately clear that the LBL-Se-NCs have superior colloidal stability compared to CS-NCs irrespective of the suspension medium. The size and zeta potential of both nanoparticles were monitored post-synthesis in RNase-free water for up to 480 h (20 days). While the size of LBL-Se-NCs started to increase slightly after 10 days due to a gradual drop in the zeta potential, their colloidal stability was markedly better than that for CS-NCs, which started to aggregate just after 2 days (Figure 2B).

Next, the siRNA release from LBL-Se-NCs and CS-NCs was monitored over time for a period of 7 days (Figure 2C). LBL-Se-NCs and CS-NCs were stored in microtubes containing 10 mM HEPES buffer (pH 7.4) and were continuously gently shaken at 37°C. At specific time points, samples were taken and centrifuged at 21,000 *g* for 60 min to determine the amount of released siRNA in the supernatant. In the case of CS-NCs, more than 50% siRNA was already released after only 8 h, reaching 100% release after just 48 h. In contrast, the siRNA release from LBL-Se-NCs was only 35% after 7 days. These results show that, under equal experimental conditions, siRNA remains better associated to LBL-Se-NCs as compared to CS-NCs.

### *In vitro* Anticancer Activity: Cell Viability and Apoptosis Assays

**FIGURE 3:**
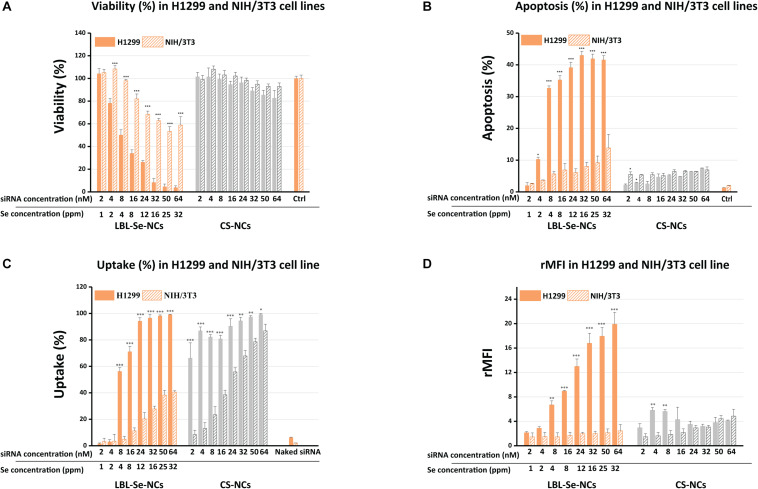
Comparison of biological effect by LBL-Se-NCs and CS-NCs in H1299 and NIH/3T3 cells. **(A)** Cell viability after treatment of H1299 (cancer cells) and NIH/3T3 (normal cells) with LBL-Se-NCs and CS-NCs. **(B)** Flow cytometric determination of apoptotic and necrotic populations of cells treated with LBL-Se-NCs in comparison with CS-NCs on H1299 and NIH/3T3 cells. **(C)** Uptake of AF647 siRNA-loaded Se-NCs and CS-NCs in H1299 and NIH/3T3 cell lines quantified *via* flow cytometry. ‘Uptake%’ refers to the percentage of cells that are positive for AF647. **(D)** The corresponding relative mean AF647 fluorescence intensity per cell (rMFI), which is proportional to the amount of particles that are taken up per cell on average. Bars represent mean ± SEM (*n* = 3, *p < 0.05; **p < 0.01; ***p < 0.001).

To determine the selective anticancer potential of LBL-Se-NCs, LBL-Se-NCs and CS-NCs were added to H1299 (cancer cells) and NIH/3T3 (normal cells) at concentrations ranging from 2 to 64 nM of siRNA, corresponding to 1–32 ppm of selenium. Note that untargeted siRNA was used in these experiments so as to judge the anticancer effect by the carriers alone. The CellTiter-Glo metabolic assay was used to quantify cell viability after 24 h. As shown in Figure 3A, LBL-Se-NCs effectively suppressed the growth of H1299 cells in a dose-dependent manner. About 50% loss in cell viability was observed at a siRNA concentration of 8 nM (4 ppm of Se) on H1299 cells, while the cell viability of NIH/3T3 normal cells was still ∼100% at the same concentration. At the highest concentration of 64 nM (32 ppm of Se), almost all H1299 cancer cells had died, while the viability of NIH/3T3 cells remained > 50%. When CS-NCs were applied to the cancer cells in the same concentration range, no clear difference in cell viability between H1299 and NIH/T3T cells could be observed, with the cell viability remaining > 80% even at the highest concentration.

Next, we proceeded to evaluate the induction of apoptosis on both cell lines. One of the methods that allow for distinguishing apoptotic cells from necrotic cells is the combination of two dyes: DiIC_1_(5) fluorochrome and PI. DiIC_1_(5) (λ_*ex*_ 638 nm; λ_em_ 658 nm) is a deep red, lipophilic dye that is selective for the mitochondria of live cells, causing high fluorescence intensity; meanwhile, for apoptotic cells, when the membrane potential of the mitochondria is no longer intact, the dye diffuses over the cytoplasm and the staining is less intense. PI (λ_*ex*_ 482 nm; λ_em_ 608 nm), cannot cross intact plasma membrane and therefore will only be present in the DNA of necrotic cells where the plasma membrane has been compromised/permeabilized. Therefore, the combination of these two dyes allows for distinguishing apoptotic from necrotic cells by flow cytometry. Treatment with LBL-Se-NCs at a concentration of 8 nM resulted in 32.0 ± 0.7% and 5.6 ± 0.8% apoptosis in H1299 and NIH/3T3 cells, respectively (Figure 3B). This means a 5.7-times higher selective apoptosis induction for the LBL-Se-NCs in cancer cells in comparison to normal cells. CS-NCs, on the other hand, had low levels of apoptosis induction in both cell lines (H1299 and NIH/3T3 cells) with no clear difference. As the CS-NCs control group includes all the substances in the LBL-Se-NCs except for the Se core, this result shows that the apoptosis effect indeed arises from the presence of the Se core. Furthermore, an obvious increase in the necrotic population could be seen in none of the treated cells, pointing to an absence of acute toxicity by either of the carriers (Supplementary Figure 2).

### *In vitro* siRNA Delivery of NCs

To verify if the cell-selective effect of LBL-Se-NCs is due to a difference in uptake, the nanocarriers were loaded with fluorescently labeled AF647 siRNA for the quantification of uptake *via* flow cytometry. Figure 3C shows that the percentage of cells positive for AF647 siRNA (uptake%) was indeed markedly higher for H1299 cells as compared to NIH/3T3 cells. The difference was even more pronounced when looking at the rMFI values, which are a measure for the amount of uptake per cell (Figure 3D). Together, these details point to a direct correlation between the amount of internalized NCs and apoptosis induction. Surprisingly, the percentage of positive cells was quite similar for both cell types when comparing CS-NCs with LBL-Se-NCs, which, at first glance, would suggest an equal amount of internalization. However, the much lower rMFI values show that this is not the case, with fairly little uptake in either of the cell lines.

### *In vitro* Transfection Efficiency of NCs

**FIGURE 4:**
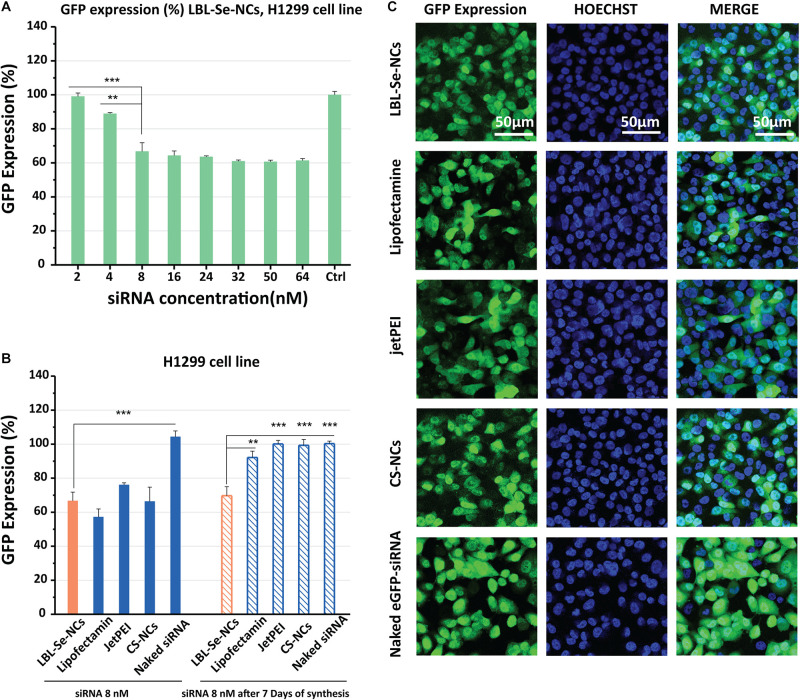
*In vitro* knockdown efficiency of LBL-Se-NCs. **(A)** GFP expression (%) of H1299 cells treated with LBL-Se-NCs as a function of siRNA concentration. **(B)** Comparison of the GFP expression level on H1299 cells after treatment with LBL-Se-NCs, Lipofectamine, jetPEI, CS-NCs, and naked siRNA, all at 8 nM effective siRNA concentration, for both freshly synthesized and 7-day-old nanocomplexes**. (C)** Confocal images of intracellular GFP knockdown of LBL-Se-NCs and control groups on H1299 cells. The scale bar corresponds to 50 μm. Data are presented as mean values ± SD (*n* = 3, *p < 0.05; **p < 0.01; ***p < 0.001).

Next, we proceeded to evaluate the ability of LBL-Se-NCs to functionally deliver siRNA into cancer cells. To this end, the transfection performance was evaluated with nanocarriers loaded with anti-eGFP siRNA in concentrations from 2 to 64 nM (Figure 4A). Briefly, H1299-eGFP cells were incubated with LBL-Se-NCs for 4 h, washed, and kept in fresh cell culture medium for an additional 24 h before eGFP knockdown with flow cytometry. The flow cytometry analysis showed that, for an effective siRNA concentration of 8 nM, the eGFP expression was reduced by ∼35%. While the eGFP expression did not reduce further at higher concentrations, cell viability did, which goes hand in hand with an increased amount of internalized nanocarriers (Figure 3A). To better appreciate the extent of the knockdown, we additionally performed a comparison with CS-NCs and popular commercial transfection reagents like jetPEI and Lipofectamine. Naked eGFP-siRNA was added as a control. As can be seen from the flow cytometry results in Figure 4B, and supported by the confocal images in Figure 4C, at the same effective siRNA concentration of 8 nM, no significant differences between LBL-Se-NCs and other carriers in terms of knockdown efficiency were observed. As expected, there was no measurable effect of the naked eGFP-siRNA. Since long-term stability and effectivity is an important consideration as well when designing nanoformulations for drug delivery, we also measured the knock-down efficiency 7 days after the various nanocomplexes were synthesized (Figure 4B). LBL-Se-NCs showed the same result as when they were freshly synthesized. This was, however, not the case for any of the other (commercial) carriers, which, apart from Lipofectamine, had completely lost their efficacy after 7 days.

### Evaluation of Endosomal Escape

**FIGURE 5:**
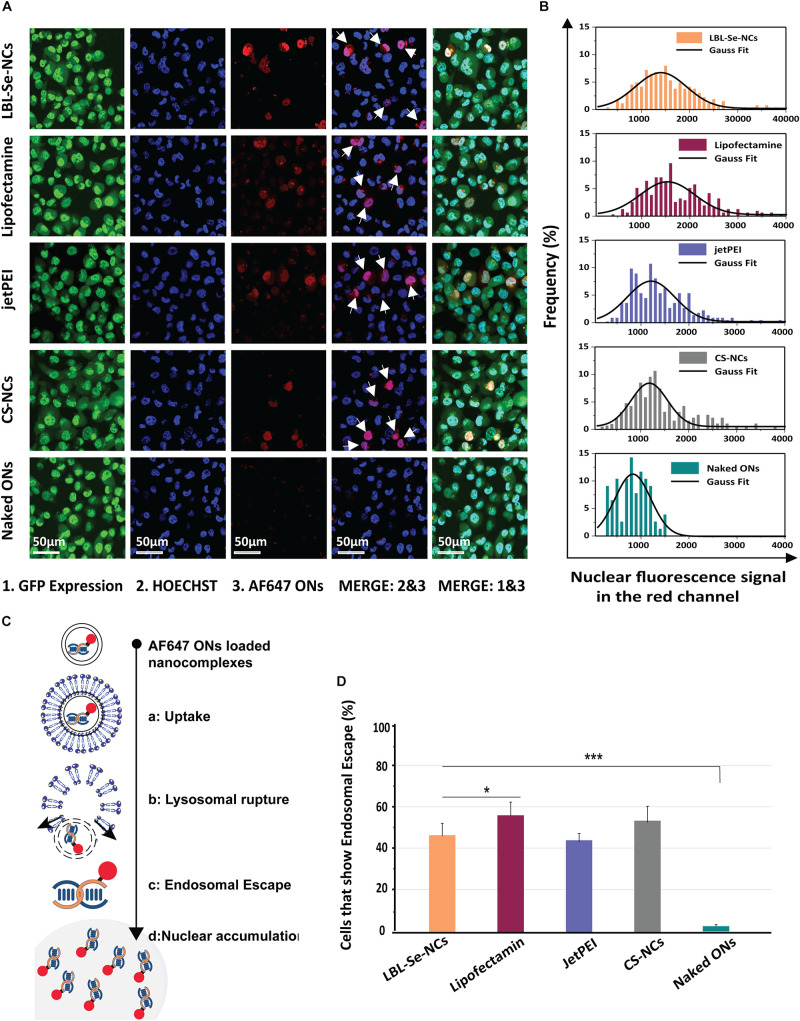
Evaluation of Endosomal Escape based on AF647 ONs. **(A)** Representative confocal images 24 h post-incubation with AF647 ONs-loaded NCs in eGFP-H1299 cells. Upon endosomal escape, the labeled ONs which are loaded into the various nanocomplexes spread toward the cytoplasm, dequench, and finally accumulate into the nucleus. Hoechst nuclei can be seen in blue, while cells in which the endosomal escape occurred show nuclear fluorescence in the red channel (white arrows). The scale bar corresponds to 50 μm. **(B)** The histogram of red fluorescent intensity of cell nuclei. **(C)** Scheme of ONs uptake, endosomal escape, and accumulation in the nucleus. **(D)** The percentage of cells that had at least one endosomal escape event as quantified from AF647 fluorescence of at least 250 nuclei. The scale bar corresponds to 50 μm, (*n* = 3, *p < 0.05; **p < 0.01; ***p < 0.001).

In order to get a deeper understanding of the experimentally observed downregulation efficiencies for eGFP, we proceeded to evaluate endosomal escape efficiencies based on a previously published fluorescence microscopy assay (Rehman et al., 2013; Vermeulen et al., 2018). Briefly, NCs were prepared with red-labeled fluorescent oligonucleotides (AF647 ONs) instead of siRNA. When incorporated into the NCs, the fluorescence of the ONs is mostly quenched. But upon endosomal escape, the labeled ONs would spread toward the cytoplasm, dequench, and the free ONs would accumulate into the nucleus (Figure 5C). As shown in the confocal images in Figure 5A, the accumulation of free ONs in the nucleus was detected in several cells, indicating that endosomal escape events had occurred for all tested NCs but not for free AF647 ONs. The histograms in Figure 5B show the red fluorescence intensity of the cell nuclei for the respective NCs as determined from the confocal images. By counting the total number of nuclei in confocal images on the one hand (by Hoechst staining) and red fluorescent nuclei on the other, the percentage of cells in which at least one endosomal escape event has occurred can be calculated. The cells that exhibited endosomal escape amounted to 43, 57, 42, and 52% for LBL-Se-NCs, Lipofectamine, jetPEI, and CS-NCs, respectively (Figure 5D). These results correlate very well to the GFP transfection efficiencies, showing that endosomal escape is an important determining factor for the transfection efficiency of these nanocarriers.

## Discussion

### Nanoparticle Characterization and Stability

In cancer nanomedicine, nanocarriers usually do not play a role in the anticancer process and only act as a vehicle to deliver their therapeutic content (Rosenblum et al., 2018). However, recent studies have shown that Se-based carriers can play a role in inhibiting cancer growth (Weekley and Harris, 2013). Relentless cell division and proliferation of cancer cells, combined with their aberrant metabolism, lead to an increased oxidized environment (Cairns et al., 2011). Therefore, to evade ROS-induced cell death, cancer cells have an aberrant metabolism that promotes some agents to scavenge ROS and repair ROS-induced damage with molecules like glutathione peroxidase, glutathione-S-transferase, glutaredoxin, thioredoxin, superoxide dismutase, and catalase (Glorieux and Calderon, 2017). This leaves the cancer cells with very little or no room to accommodate any further increase in ROS levels. Indeed, even a slight increase in ROS levels can lead to apoptosis-dependent cell death in the cancer cells (Cairns et al., 2011). This is not the case for normal healthy cells, which can still cope with increased ROS levels *via* the upregulation of antioxidant systems. On the other hand, this oxidative stress and redox imbalance in cancer cells will stimulate the formation of Se–S adducts from Se compounds and, subsequently, will increase receptor-mediated uptake of Se in cancer cells, in contrast to normal cells (Olm et al., 2009; Mandal et al., 2010). Hence, ROS induction may selectively kill cancer cells without affecting normal cells (Gorrini et al., 2013). Consequently, inducing oxidative stress in cancer cells using redox modulators provides an interesting therapeutic opportunity for cancer treatment (Gandin et al., 2018). Se compounds have been well documented to act as redox modulators and have shown higher selectivity and sensitivity in apoptosis induction in the malignant cells (Valdiglesias et al., 2010; Chaiswing et al., 2018). In this regard, approaches based on Se nanocarriers have shown promising anti-proliferation activity and inhibition of cancer cells, exhibiting different effects in malignant vs. normal cells. Moreover, the use of SeNPs conjugated with siRNAs has shown great promise in many types of cancer research (Zheng et al., 2015; Li et al., 2016). Despite these demonstrated advantages, the application of Se-based carriers for gene delivery remains hindered due to poor stability (Yu et al., 2014; Li et al., 2016; Xia et al.,2020a,b). The exact anticancer mechanisms of SeNPs for the formulation presented in this study still need to be explored. Nevertheless, it is well documented that the anticancer properties of Se are related to the induction of apoptosis (Huang et al., 2013; Liao et al., 2015; Tian et al., 2020). In this study, we proposed to take advantage of these properties and set out to develop a novel Se-based siRNA delivery system that combines the benefits of Se-based carriers with improved colloidal stability while still providing efficient siRNA transfections.

We designed a layer-by-layer strategy to coat Se@CS in such a way that the core confers a solid anchoring nucleus around which the layer-by-layer system is built. By using UV-Vis spectroscopy and agarose gel electrophoresis, the ability of chitosan to interact with siRNA was confirmed. A third and final chitosan layer was needed, however, to have a fully stable formulation that can withstand aggregation during centrifugation washing steps. This suggests that the surface charge in this system is an important determinant of the stability of LBL-Se-NC. We would like to note, however, that the nature of the third layer deposition cannot be simply electrostatic due to the lack of net negative zeta potential, but it is possible that local areas of high electron density groups facilitate the attachment of the third layer of chitosan. The increase in the size and positive surface charge after applying the third layer definitely indicates that chitosan deposition has been performed (Figures 1C,D). Furthermore, in Figure 1E, after the formation of the third layer with chitosan, an increase of around 200 nm at the extinction spectrum was observed, which is related to the attached chitosan on the surface. The final chitosan layer is also visible in the TEM images as a faint ‘halo,’ which resulted in an effective increase in the overall size of the nanoparticle to 95 ± 11 nm (Figure 1G). How this interaction took place even between positively charged macromolecules is a point that seems to be related to the spatial and structural shape of the polymers that were also reported in previous studies (Wu et al., 2019). Importantly, we found that the presence of a Se resulted in markedly enhanced stability over time, showing no variation in size and zeta for at least 20 days (Figure 2). Instead, carriers made from CS alone started to aggregate just after 48 h.

### Efficacy and Biological Effect of NCs

When applied to H1299 cells, LBL-Se-NCs had a clear toxic effect, which was not observed when the cells were treated with siRNA chitosan complexes without Se core (CS-NCs). Furthermore, LBL-Se-NCs were found to selectively induce apoptosis in H1299 but not, or much less, in NIH/3T3 normal cells. Also, the uptake levels of LBL-Se-NCs in NIH/3T3 cells were remarkably lower than those in H1299 cells. Altogether, the selective anticancer effect of the LBL-Se-NCs in H1299 cancer cells could be confirmed, which can be directly linked to apoptosis induction in cancer cells by Se.

In addition to this selective apoptosis, we evaluated the intracellular delivery of the developed LBL-Se-NCs in this study by evaluating the eGFP knockdown efficacy in H1299 cells. We found that LBL-Se-NCs induced similar knockdown levels as commercial transfection reagents like jetPEI and Lipofectamine, which confirms their high transfection efficiency. Interestingly, transfection efficiencies could be perfectly correlated with the endosomal escape efficiency of the various NCs (Figures 5A–D). Furthermore, and importantly, unlike the commercial transfection reagents or CS-NCs, LBL-Se-NCs were still as functional as just after synthesis even after 7 days, which is yet another demonstration of the superior stability of this system.

## Conclusion

Herein, LBL-Se-NCs were successfully prepared using a simple layer-by-layer electrostatic assembly of siRNA and chitosan on Se@CS core nanoparticles. The first layer of chitosan functioned as an anchoring substrate for siRNA. A third and final CS layer was added as a protective layer, which provided outstanding colloidal stability for at least 20 d, which showed no variation in size or zeta potential irrespective of the suspension medium. Furthermore, the final CS layer ensured stable siRNA complexation with only 35% release 7 days post-synthesis, which was significantly better than CS-NCs. LBL-Se-NCs were found to selectively induce 32% apoptosis in H1299 cancer cells, which was 5.7 times more than that in NIH/3T3 normal cells. We could prove that this effect is linked to the presence of the Se core, since CS-NCs showed low levels of apoptosis in both cancerous and normal cells. LBL-Se-NCs were also found to be able to induce 35% downregulation of eGFP in H1299 cells, which was the case both just after synthesis and 7 days post-synthesis. This clearly showed the superior stability of the presented LBL nanocarriers herein, as all the other carriers (i.e., CS-NCs, jetPEI polyplexes, and Lipofectamine lipoplexes) completely lost their efficacy over time.

In conclusion, the induction of selective apoptosis and the improved siRNA silencing over extended periods of time make LBL-Se-NCs an excellent candidate as an off-the-shelf formulation with a dual mode of action against cancer cells.

## Data Availability Statement

The original contributions presented in the study are included in the article/Supplementary Material, further inquiries can be directed to the corresponding author/s.

## Author Contributions

MS presented the initial idea for the research, planned the experiments of this study, and wrote most of the manuscript. MS and ES conducted most of the experiments including the synthesis and the characterization part. KB, JF, and RF-M supervised the experiment and advised on data analysis and writing. SD reviewed the data and the results. ZS advised on experiments and data analysis. HD provided the overall guidance and help for the use of confocal microscopy. RD performed the sample preparation for TEM and recorded the TEM images. All authors contributed to the article and agreed on the submitted version.

## Conflict of Interest

The authors declare that the research was conducted in the absence of any commercial or financial relationships that could be construed as a potential conflict of interest.

## Abbreviations

Se: selenium
CS: chitosan
NPs: nanoparticles
NCs: nanocomplexes
SeNPs: selenium nanoparticles
LBL-Se-NCs: layer-by-layer selenium nanocomplexes
CS-NCs: chitosan nanocomplexes.
