# Supplementary material for: Enhanced siRNA Delivery and Selective Apoptosis Induction in H1299 Cancer Cells by Layer-by-Layer-Assembled Se Nanocomplexes: Toward More Efficient Cancer Therapy
Source: Front Mol Biosci. 2021 Apr 20;8:639184. doi: 10.3389/fmolb.2021.639184 (PMC8093573; doi:10.3389/fmolb.2021.639184)
Supplement: Supplementary file 1 [file Data_Sheet_1.PDF]

Supplementary Material

1 Supplementary Figures

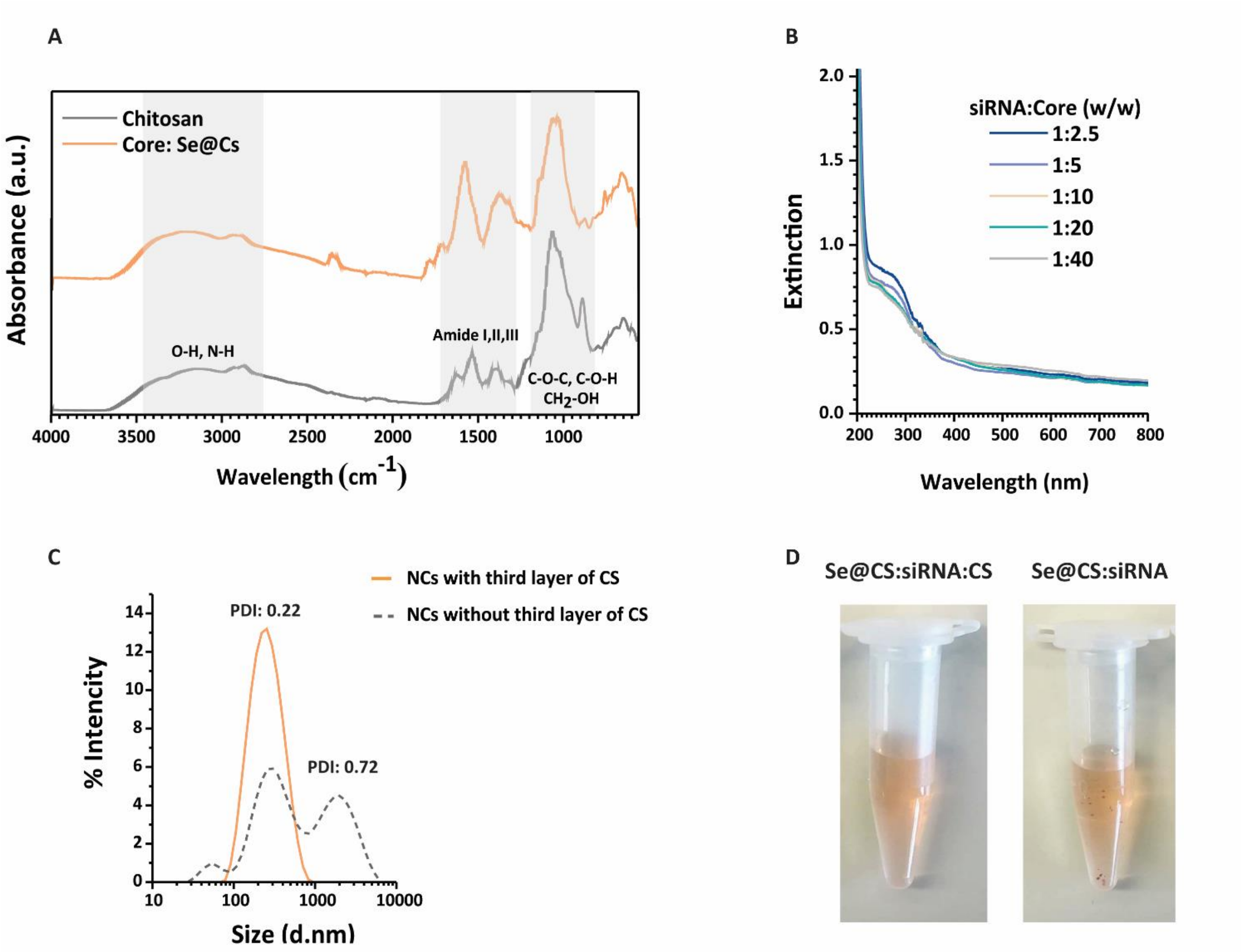

Supplementary Figure 1. (A) FT-IR spectra of chitosan and Se@CS. **(B)** The siRNA loading capacity of Se@CS nanoparticles from 1:2.5 to 1:40 siRNA:core mass ratios were evaluated by measuring the remaining siRNA content in the supernatant by UV-vis spectra. **(C)** DLS of NCs with and without the third chitosan layer. **(D)** Difference in NCs's resuspension in ddi. Water after centrifugation with and without the third chitosan layer.

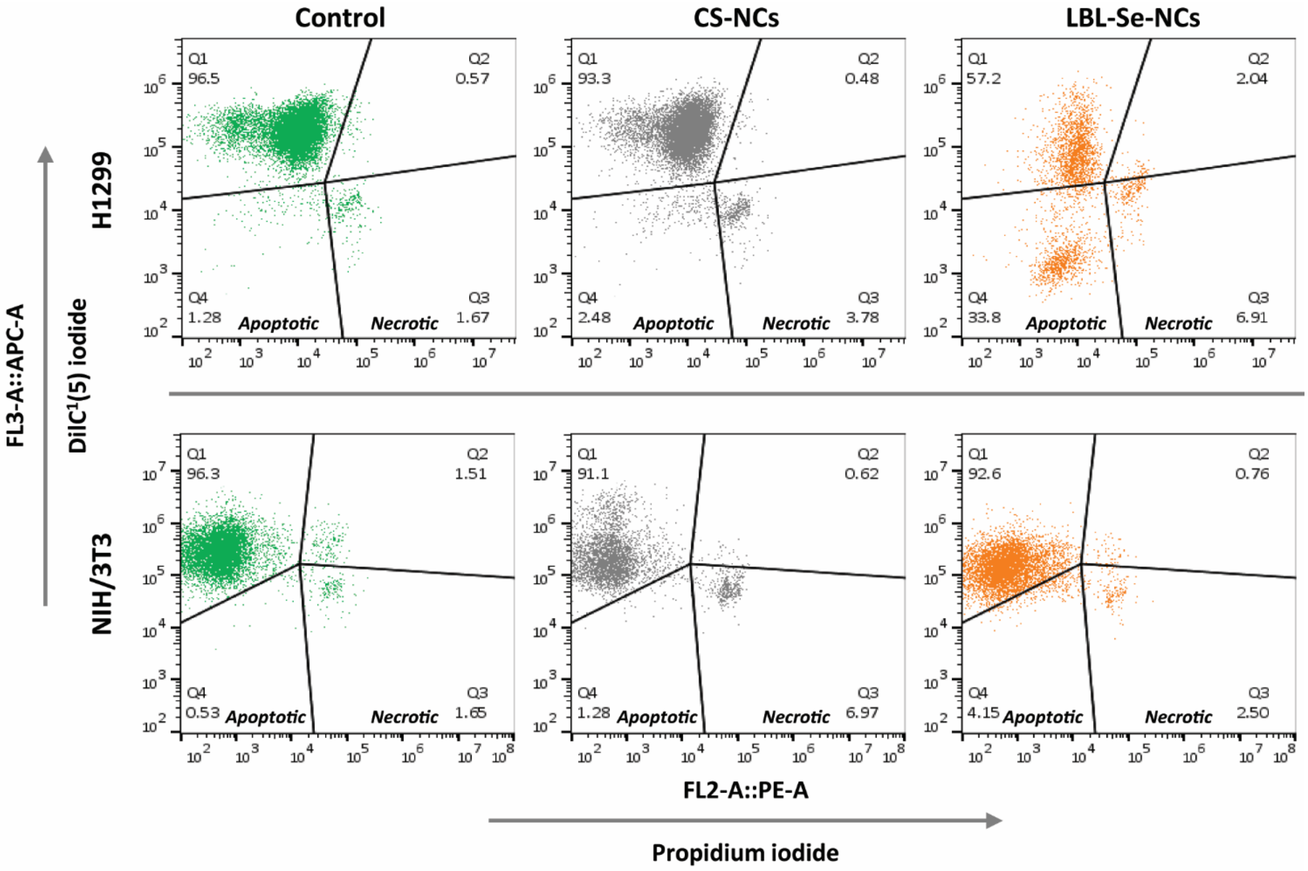

Supplementary Figure 2. Determination of apoptotic and necrotic populations of cells treated with LBL-Se-NCs in comparison with CS-NCs on H1299 and NIH/3T3 cells was performed using Propidium iodide (PI) and dihexaoxacarbocyanine iodide (DiIC1) dyes and flow cytometry analysis. Dot plots obtained from the flow cytometry quantification of cells treated with 8 nM LBL-Se-NCs and CS-NCs.
